# Role of Norepinephrine in IL-1β-Induced Chondrocyte Dedifferentiation under Physioxia

**DOI:** 10.3390/ijms20051212

**Published:** 2019-03-11

**Authors:** Saskia Speichert, Natalie Molotkov, Karima El Bagdadi, Andrea Meurer, Frank Zaucke, Zsuzsa Jenei-Lanzl

**Affiliations:** Dr. Rolf M. Schwiete Research Unit for Osteoarthritis, Orthopaedic University Hospital Friedrichsheim gGmbH, Marienburgstr. 2, 60528 Frankfurt/Main, Germany; saskia.speichert@web.de (S.S.); natalie.molotkov@friedrichsheim.de (N.M.); karima.elbagdadi@friedrichsheim.de (K.E.B.); andrea.meurer@friedrichsheim.de (A.M.); frank.zaucke@friedrichsheim.de (F.Z.)

**Keywords:** osteoarthritis, chondrocytes, dedifferentiation, norepinephrine, interleukin-1β, adrenoceptors, physioxia

## Abstract

As part of the pathogenesis of osteoarthritis (OA), chondrocytes lose their phenotype and become hypertrophic, or dedifferentiate, mainly driven by interleukin-1β (IL-1β). The contribution of other factors to the dedifferentiation process is not completely understood. Recent studies suggested a dose-dependent role for the sympathetic neurotransmitter norepinephrine (NE) in OA chondrocyte metabolism. Therefore, the aim of this study was to analyze the contribution of NE (10^−8^ M, 10^−6^ M) to human articular OA chondrocyte dedifferentiation in the absence or presence of IL-1β (0.5 ng/mL). Here, we demonstrate that OA chondrocytes express α2A-, α2C- and β2-adrenoceptors (AR) and show the characteristic shift towards a fibroblast-like shape at day 7 in physioxic monolayer culture. NE alone did not affect morphology but, in combination with IL-1β, markedly accelerated this shift. Moderate glycosaminoglycan (GAG) staining was observed in untreated and NE-treated cells, while IL-1β strongly decreased GAG deposition. IL-1β alone or in combination with NE decreased SOX9, type II collagen, COMP, and aggrecan, and induced MMP13 and ADAMTS4 gene expression, indicating an accelerated dedifferentiation. NE alone did not influence gene expression and did not modulate IL-1β-mediated effects. In conclusion, these results indicate that low-grade inflammation exerts a dominant effect on chondrocyte dedifferentiation and should be targeted early in OA therapy.

## 1. Introduction

Osteoarthritis (OA) is one of the most common painful and disabling chronic degenerative joint diseases worldwide [1,2]. Besides ageing and obesity, further OA risk factors have been identified during the past decades such as genetics, gender, diet, injury, or overloading of the joints [3]. However, there are still many deficits in our understanding of the exact pathophysiology of OA at the molecular level. During OA progression, the articular chondrocyte phenotype characterized by high type II collagen and proteoglycan expression becomes unstable due to hypertrophic terminal differentiation or dedifferentiation [4,5,6]. Hypertrophic chondrocytes start to produce type X collagen and the type II collagen-degrading enzyme MMP-13, whereas dedifferentiating chondrocytes display a fibroblast-like morphology with high type I collagen production and concomitant down-regulation of type II collagen, aggrecan, and cartilage oligomeric matrix protein (COMP) expression [7,8,9]. Articular cartilage-derived chondrocytes cultured in monolayer dedifferentiate rapidly and show the characteristic morphological shift towards a fibroblast-like shape, as well as changes in extracellular matrix (ECM) synthesis as described above [10]. Therefore, monolayer chondrocyte culture can be used as an in vitro model of chondrocyte dedifferentiation allowing analysis of the effect of different factors on the dedifferentiation process. Chondrocyte dedifferentiation in OA cartilage tissue is primarily induced by pro-inflammatory mediators such as IL-1β, the main pro-inflammatory cytokine in OA [11,12]. IL-1β has been detected in synovial fluid of OA patients at concentrations of 1.25–100 pg/mL [13,14], representing a low level of inflammation compared to other chronic degenerative and inflammatory joint diseases like rheumatoid arthritis, in which IL-1β levels can reach up to 15 pg/mL, accompanied by very high TNF-α concentrations [15]. However, other factors that might contribute, as well as underlying molecular mechanisms inducing chondrocyte dedifferentiation in OA, are still largely unknown. During the last few decades, the first evidence emerged that besides aging, genetics, trauma, and inflammation, endocrine and sympathetic nervous factors also influence cartilage homeostasis, regeneration capacity, and OA development [16]. Sympathetic nerve fibers are characterized by high tyrosine hydroxylase (TH) expression and activity [17]. This enzyme is responsible for the conversion of tyrosine to L-DOPA, the first rate-limiting step in the biosynthesis of catecholamines such as dopamine (DA), NE, and epinephrine (E). Recently, TH-positive cells and nerve fibers were detected in synovial tissue and increased levels of NE were found in the synovial fluid of knee trauma and OA patients, whereas DA and E were not detectable [18]. It is also known that OA articular chondrocytes express adrenoceptors (ARs), suggesting a role for the sympathetic nervous system in OA pathogenesis [19]. Recent studies demonstrated that NE can modulate IL-1β-induced changes in chondrocyte metabolism mediated by different AR subtypes depending on the concentration of NE [19]. Due to the affinity of the distinct ARs, NE in low concentrations (≤10^−7^ M) primarily acts via αARs, while βARs are preferentially activated at high NE concentrations (≥10^−7^ M) [20,21]. However, these studies were performed under 20% O_2_ concentration, representing a hyperoxic and unphysiological condition for articular chondrocytes, because O_2_ concentrations in cartilage tissue lie between 1–3% (physioxia) [22]. Interestingly, it has been shown recently that physioxia alone leads to a deceleration of the dedifferentiation process. An upregulation of SOX9 and COL2A1 under physioxic conditions led to a prolonged stabilization of the chondrocytic phenotype [23].

Therefore, the aim of this study was to analyze for the first time the contribution of NE to the dedifferentiation response of human articular OA chondrocytes in the absence or presence of low-dose IL-1β under physioxic conditions. Based on the studies described above, we hypothesized that NE treatment accelerates chondrocyte dedifferentiation and might potentiate the effects of IL-1β on the dedifferentiation process. The results will provide new insight into OA pathophysiology and might unravel potential novel therapeutic options for OA treatment.

## 2. Results

### 2.1. Chondrocyte Dedifferentiation under Physioxia

**Figure 1:**
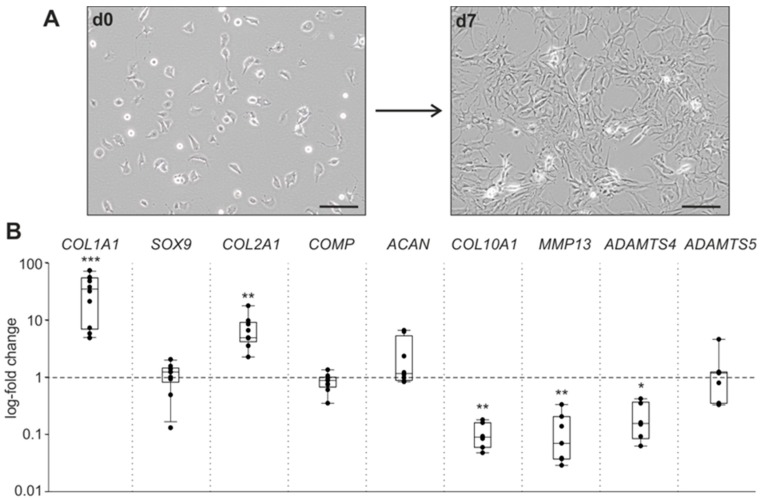
Chondrocyte phenotype shift during dedifferentiation under physioxia. (**A**) Change of human osteoarthritis (OA) chondrocytes from a polygonal to a fibroblast-like morphology after seven days under physioxic conditions (representative images of chondrocytes isolated from one OA patient, magnification 200×, scale bars represent 100 μm). (**B**) Gene expression changes of the transcription factor SOX9, ECM components, and ECM-degrading enzymes after seven days in monolayer culture under physioxic conditions compared to day 0 (gene expression on day 0 = 1, represented by the dashed line). All experiments were carried out with cells from 8–10 patients, using duplicates for each patient. Each circle shows the mean of the duplicates. Data are presented as box plots, where the boxes represent the 25th to 75th percentiles, the lines within the boxes represent the median, and the lines outside the boxes represent the 10th and 90th percentiles. Significant *p*-values against day 0 are presented as: * (*p* ≤ 0.05), ** (*p* ≤ 0.01), *** (*p* ≤ 0.001).

First, morphological changes and alterations in the expression of ECM-related genes in OA chondrocytes after seven days in monolayer culture were investigated. One day after isolation, chondrocytes attached to the culture plate and exhibited a round and polygonal morphology (Figure 1A). After seven days in monolayer under physioxia, chondrocytes dedifferentiated and showed the characteristic shift towards a fibroblast-like shape (Figure 1A). Gene expression levels of COL1A1 and COL2A1 increased, while COL10A1, matrix metallopeptidase 13 (MMP13), and ADAM metallopeptidase with thrombospondin type 1 motif 4 (ADAMTS4) expression decreased during dedifferentiation, compared to chondrocytes at day 0 (COL1A1, *p* < 0.001; COL2A1, *p* = 0.007; COL10A1, *p* = 0.007; MMP13, *p* = 0.008; ADAMTS4, *p* = 0.014). The mRNA expression of the transcription factor SOX9, as well as of the ECM components COMP and aggrecan (ACAN), remained stable until day 7 (Figure 1B). No age- or gender-dependent differences were observed regarding chondrocyte dedifferentiation under physioxia.

### 2.2. AR and IL-1βR Expression Profile of Human Articular Chondrocytes

**Figure 2:**
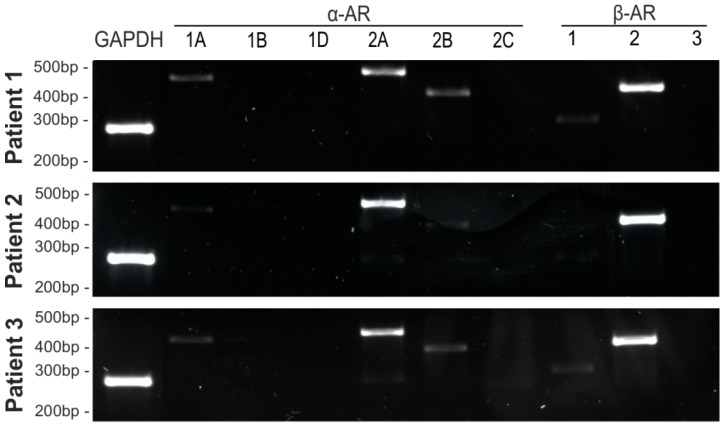
Adrenergic receptor profile of OA chondrocytes. Gene expression of different adrenoceptor subtypes in untreated primary chondrocytes isolated from three patients at day 0 (representative images of three OA donor cell isolations).

Next, the expression profile of ARs and the IL-1β receptor (IL-1βR) in OA chondrocytes was examined, because the presence of distinct receptors is the prerequisite for NE or IL-1βR action. Freshly isolated OA chondrocytes expressed distinct α- and β-AR subtypes (Figure 2) as well as the IL-1βR (Supplementary Figure S2). Gene expression levels of β2-AR and IL-1βR were the highest, α1A-AR was moderately expressed, and α2A-AR gene expression was only weakly detectable on day 0. The receptors α1A-, α2A-, α2B-, α2C-, and β1-AR were not detected, and nor was the TH gene (Figure 2). No age- or gender-dependent differences were observed regarding AR or IL-1βR expression in chondrocytes.

### 2.3. NE-Induced Intracellular Signaling in Human Articular Chondrocytes

**Figure 3:**
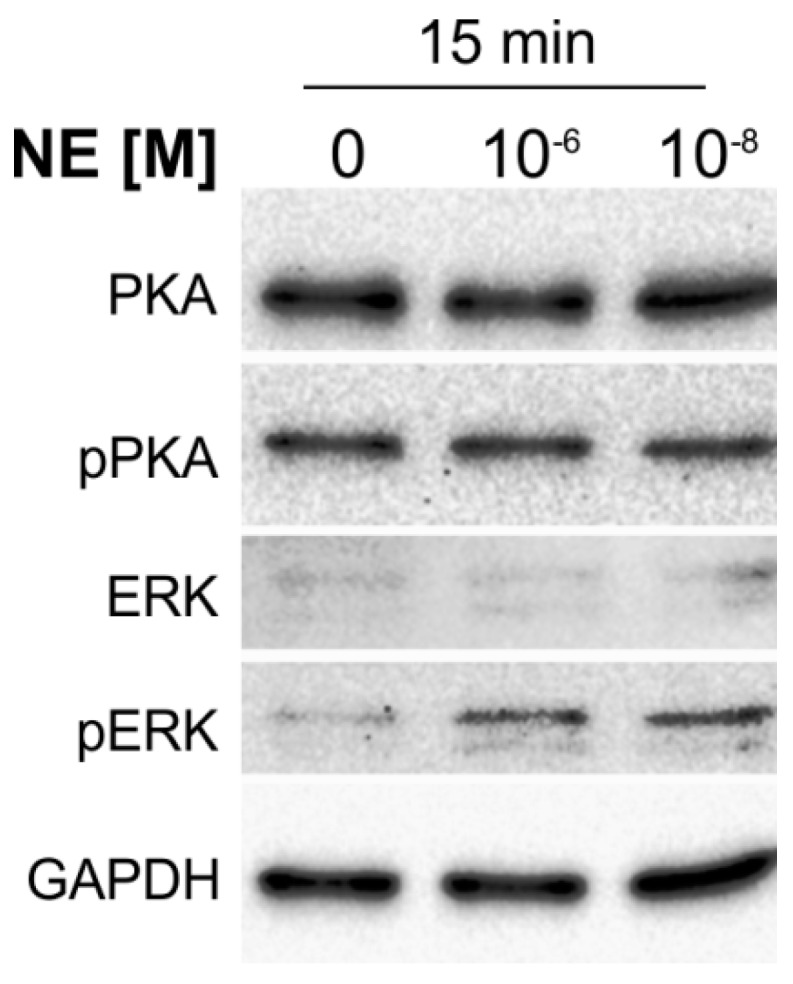
NE-mediated activation of intracellular signaling pathways in chondrocytes. No changes in PKA phosphorylation are visible, while both low and high NE concentrations increase phosphorylation of ERK after 15 min (representative images of cells isolated form one OA patient).

Chondrocytes were treated for 15 min with NE (10^−8^ or 10^−6^ M) and the activation of the two major AR-mediated signaling pathways, the ERK1/2 and PKA pathways, were analyzed. Both low and high NE concentrations induced ERK1/2 phosphorylation after 15 min, while PKA phosphorylation was not increased by any treatment (Figure 3).

### 2.4. Effects of NE and IL-1β on Chondrocyte Morphology and Viability during Dedifferentiation

**Figure 4:**
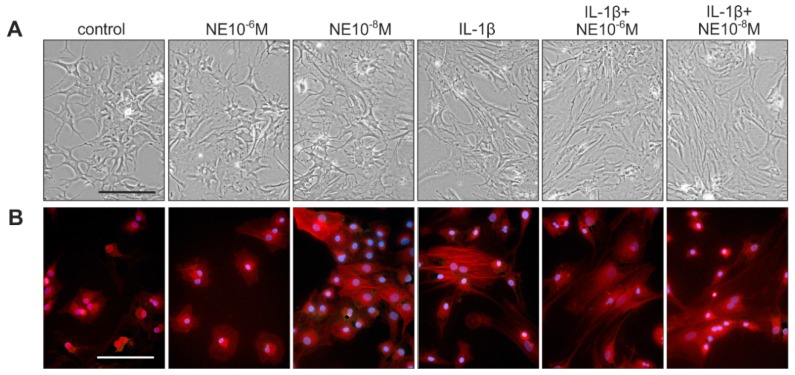
Effects of NE and IL-1β on chondrocyte morphology and cytoskeleton organization. (**A**) Phase contrast microscopic appearance of OA chondrocytes at day 7 after treatment with NE, IL-1β, or NE + IL-1β (representative images of cells isolated form one OA patient, magnification 200×). (**B**) Phalloidin staining of the actin cytoskeleton at day 7 after treatment with NE, IL-1β, or NE + IL-1β (representative images of cells isolated form one OA patient, magnification 200×, red: actin, blue: nuclei). Scale bars represent 100 μm.

After seven days in culture, untreated chondrocytes showed the characteristic morphological changes towards a fibroblastoid shape, as detected by phase contrast microscopy and actin cytoskeleton staining (Figure 4A,B). Chondrocytes treated with NE alone (10^−6^ M and 10^−8^ M) did not differ morphologically from controls (Figure 4A,B). IL-1β treatment resulted in an accelerated morphological shift with more elongated cells (Figure 4A) and clearly visible phalloidin-stained stress fibers (Figure 4B), as described earlier [10,24]. Similarly, chondrocytes incubated with a combination of IL-1β and NE showed a morphology comparable to the IL-1β-treated group, with pronounced elongation of the cell shape (Figure 4A) and strong staining of actin stress fibers (Figure 4B). The age or the gender of patients did not influence the NE- and IL-1β-mediated changes in chondrocyte morphology and viability during dedifferentiation.

Compared to untreated control, neither NE or IL-1β treatment, nor combined NE and IL-1β treatment, changed cell viability of chondrocytes (Supplementary Figure S1).

### 2.5. NE- and IL-1β-Mediated Effects on Gene Expression

**Figure 5:**
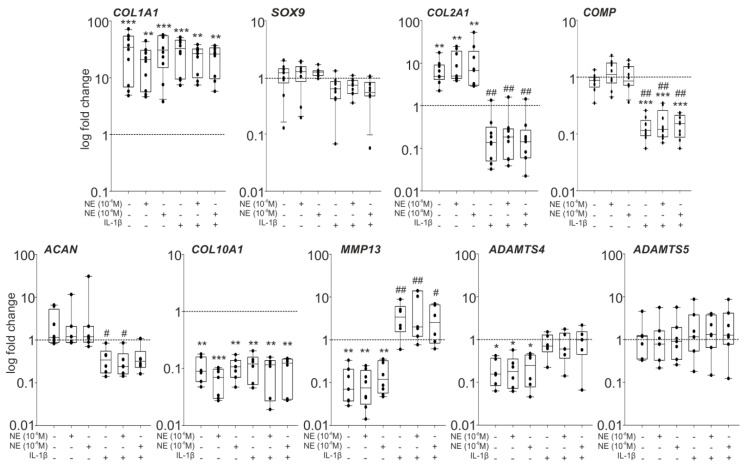
Effects of NE and IL-1β on OA chondrocyte gene expression. Gene expression changes of the transcription factor SOX9, ECM components, and ECM-degrading enzymes after seven days in monolayer culture under NE and/or IL-1β influence and physioxic conditions compared to day 0 (gene expression on day 0 = 1, represented by the dashed line). All experiments were carried out with cells from 8–10 patients, using duplicates for each patient. Each circle shows the mean of duplicates per patient. Box plots are explained in legend to Figure 1. Significant *p*-values against day 0 are presented as: * (*p* ≤ 0.05), ** (*p* ≤ 0.01), *** (*p* ≤ 0.001). Significant *p*-values against untreated control are presented as: ^#^ (*p* ≤ 0.05), ^##^ (*p* ≤ 0.01).

Compared to the untreated control with significantly (*p* < 0.001) elevated COL1A1 gene expression after seven days, no changes in COL1A1 expression were detected in chondrocytes incubated with low or high NE concentrations, with IL-1β alone, or with NE and IL-1β (Figure 5). SOX9 expression was not influenced by NE treatment compared to untreated control group at day 7, while IL-1β alone or in combination with NE slightly decreased the SOX9 level. Similarly, COL2A1 was not affected by NE treatment compared to day 7 control, but IL-1β alone or in combination with NE caused a strong and significant (*p* = 0.006) decrease of COL2A1. No changes in COMP gene expression of chondrocytes were detectable after incubation with NE, however, treatment with IL-1β alone or in combination with NE resulted in significantly (*p* = 0.006) reduced COMP levels (*p* < 0.001 to day 0). Compared to untreated control, ACAN expression was not influenced by NE, but IL-1β alone or in combination with 10^−6^ M NE reduced ACAN gene expression (IL-1β to control *p* = 0.039, IL-1β + 10^−6^ M NE to control *p* = 0.015). Neither NE or IL-1β alone, nor the simultaneous treatment with NE and IL-1β, caused any effect on the reduced COL10A1 gene expression of untreated control chondrocytes after seven days. The decreased MMP13 level of untreated chondrocytes at day 7 was not affected by NE treatment, while IL-1β alone or in combination with NE significantly (IL-1β to control *p* = 0.006, IL-1β + 10^−6^ M NE to control *p* = 0.002, IL-1β + 10^−8^ M NE to control *p* = 0.007) induced MMP13 gene expression. Gene expression of the aggrecanase ADAMTS4 did not change after NE treatment compared to untreated control group at day 7, but increased when chondrocytes were incubated with IL-1β alone or with IL-1β in combination with NE (IL-1β to control *p* = 0.026, IL-1β + 10^−6^ M NE to control *p* = 0.026, IL-1β + 10^−8^ M NE to control *p* = 0.041). In contrast, ADAMTS5 gene expression remained unchanged; neither NE or IL-1β alone, nor the simultaneous treatment with NE and IL-1β, caused any effect on ADAMTS5 gene expression (Figure 5). The age or the gender of patients did not influence the NE- and IL-1β-mediated changes in chondrocyte gene expression during dedifferentiation.

### 2.6. NE- and IL-1β-Mediated Effects on ECM Deposition

**Figure 6:**
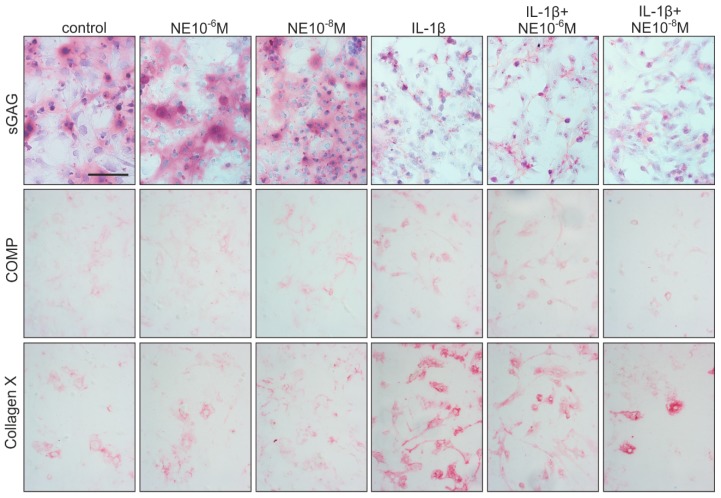
Effects of NE and IL-1β on ECM deposition. DMMB staining of sGAGs and immunohistochemical detection of COMP and type X collagen in OA chondrocyte cultures after seven days under NE or/and IL-1β influence and physioxic conditions (representative images of cells isolated form one OA patient). Scale bar represents 100 μm.

DMMB (1,9-dimethyl-methylene blue) staining of sulphated glycosaminoglycans (sGAGs) after seven days in monolayer culture showed a clearly visible GAG deposition in the untreated control group (Figure 6). Treatment of chondrocytes with low or high NE concentration did not result in any differences regarding GAG deposition (Figure 6). In contrast, chondrocytes treated with IL-1β alone, or with IL-1β in combination with NE, produced strongly reduced sGAG amounts (Figure 6). Immunohistochemical staining of COMP did not show differences between untreated controls, NE-treated groups, and IL-1β- or IL-1β plus NE-treated chondrocytes (Figure 6). Collagen X deposition in NE-treated groups did not differ from untreated controls, but chondrocytes treated with IL-1β alone or with IL-1β in combination with NE showed a more intense staining for type X collagen (Figure 6). The age or the gender of patients did not influence the NE- and IL-1β-mediated changes in ECM deposition during dedifferentiation.

### 2.7. Changes in AR Expression during Dedifferentiation

**Figure 7:**
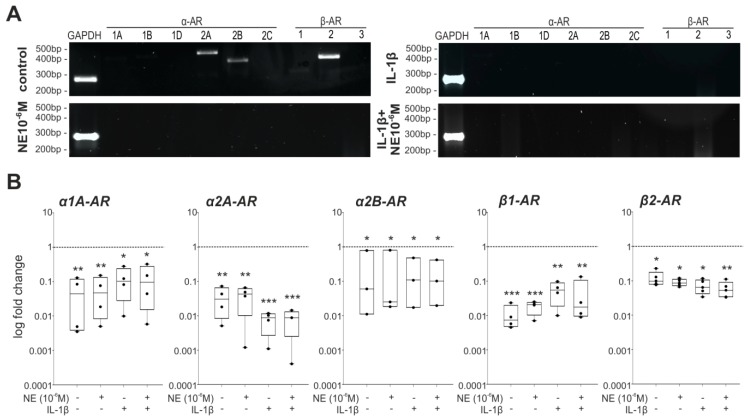
Adrenergic receptor profile of OA chondrocytes after seven days in monolayer culture. (**A**) Gene expression of different adrenoceptor subtypes in untreated or NE- and/or IL-1β-treated primary chondrocytes at day 7 (representative images of cells isolated form one OA patient). (**B**) Quantitative PCR analysis of expression levels of adrenoceptor subtypes at day 0 and after seven days without treatment as well as under NE or/and IL-1β influence and physioxic conditions. Significant *p*-values against day 0 are presented as: * (*p* ≤ 0.05), ** (*p* ≤ 0.01), *** (*p* ≤ 0.001).

Expression of detected ARs significantly decreased after seven days of monolayer culture in untreated control groups and in NE-treated groups compared to freshly isolated chondrocytes at day 0. IL-1β alone or in combination with NE significantly inhibited the expression of ARs (Figure 7, *p*-values are indicated). In contrast, no changes in IL-1βR gene expression were observed (Supplementary Figure S2). Neither NE nor IL-1β alone or in combination with NE influenced IL-1βR expression compared to day 0 chondrocytes and to untreated controls at day 7 significantly (Supplementary Figure S2).

## 3. Discussion

The pathogenesis of OA, a chronic degenerative and secondary inflammatory whole joint disease [25,26], is influenced by numerous factors, such as biomechanics, age, gender, genetics, and diet [27]. The first pathologic changes arise in the articular cartilage tissue [26]. At the cellular level, processes like chondrocyte clustering or apoptosis, hypertrophy, and dedifferentiation are involved in OA progression [12,26]. The major factor inducing chondrocyte dedifferentiation is the pro-inflammatory cytokine IL-1β [12]. During the past decade, the role of the sympathetic nervous system, in particular the role of NE, in manifestation and progression of OA has attracted increasing attention. However, studies describing mostly catabolic effects of NE in chondrocytes were performed under hyperoxic conditions and did not focus on the process of chondrocyte dedifferentiation [19]. Moreover, the effect of OA-specific low-grade inflammation alone or in combination with NE has never been investigated in this context. Therefore, we analyzed, for the first time, the influence of NE on monolayer chondrocytes in the presence and absence of IL-1β under physioxic condition, with special regard to the dedifferentiation process.

As a first step, human articular chondrocytes obtained from OA patients were cultivated in monolayer culture under physioxia (2% O_2_) for seven days in order to initiate the dedifferentiation process in vitro [10]. Most existing studies dealing with chondrocyte dedifferentiation chose much longer culture periods or even repetitive passaging [28,29]. The gene expression profile of chondrocytes losing their phenotype during long-term dedifferentiation is well characterized under hyperoxic conditions (20% O_2_): Expression of hyaline cartilage matrix-specific genes such as SOX9, COL2A1, ACAN, COMP, and COL10A1 massively decreased [30,31,32], whereas COL1A1, characteristic for fibrocartilage, greatly increased [9,10]. In addition, gene expression of the matrix-degrading enzymes MMP-13, ADAMTS4, and ADAMTS5 is reduced in chondrocytes dedifferentiated under hyperoxia at passage four [10]. Under physioxia, the long-term dedifferentiation process is decelerated, as characterized by a lesser increase in COL1A1 and unchanged or increased SOX9, COL2A1, and ACAN genes [23,33]. In the present study, chondrocytes already showed the characteristic morphological shift towards a fibroblast-like shape after seven days under physioxia [10,34,35]. In contrast to morphological changes, the expression of hyaline cartilage matrix-specific genes was not as strongly altered in monolayer culture after seven days under physioxia: COL1A1gene expression increased, COL10A1, MMP-13, and ADAMTS4 decreased, COL2A1 increased, and SOX9, COMP, ACAN, and ADAMTS5 gene expression remained unchanged, suggesting that our physioxic chondrocyte culture was in an intermediate stage after seven days in monolayer [36]. However, the dedifferentiation process was initiated and we believe that seven days in physioxic chondrocyte monolayer culture is an adequate condition to monitor the effects of specific treatments potentially affecting or modulating the dedifferentiation process. We therefore treated freshly isolated chondrocytes for the first time with IL-1β and NE and performed our analysis after seven days of culture so as to not miss regulatory events within this early phase of the dedifferentiation process.

It has been reported earlier that NE and IL-1β are present in the synovial fluid of OA patients in physiological or pathophysiological concentrations, respectively [13,18]. However, the expression of the corresponding specific ARs, as well as the IL-1βR on the target cell, is a prerequisite for their action. In freshly isolated chondrocytes, we could confirm the expression of different AR subtypes as well as of the IL-1βR. The ARs α1A-, α2A-, β2-AR, and the IL-1βR, were strongly expressed, while α2B- and α2C-AR expression was weak. α1B-, α1D-, α2B-, and β3-AR could not be detected at mRNA level. The only comparable study analyzing AR expression in OA chondrocytes was performed by Lorenz et. al. [19] and they also found strongly expressed α2A- β2-AR, but no α1A-AR. One reason for the lack of α1A-AR expression in Lorenz’s study could be that hyperoxic culture conditions might affect AR expression [37]. Furthermore, earlier studies described that OA articular chondrocytes express IL-1βR, which has been now confirmed by our study [12]. In order to consider possible autocrine effects, gene expression of TH was analyzed. In contrast to Lorenz et al. [19], TH was not detectable in our freshly isolated OA chondrocytes, therefore, autocrine effects can be excluded.

Since NE usually exhibits opposite dual effects via different α- and βARs, two relevant NE concentrations were tested in our monolayer model in presence or absence of IL-1β. Interestingly, NE alone, in low or in high concentration, affected neither chondrocyte morphology, ECM-specific gene expression, nor ECM deposition, although other studies showed clear inhibition of ECM synthesis [38] as well as proliferative or apoptotic effects [19]. One possible explanation is that these authors focused on three-dimensional redifferentiation processes after dedifferentiation and not exclusively on chondrocyte dedifferentiation in monolayer culture.

Though the strong dedifferentiation-inducing effects of IL-1β are well-known, most previous studies used extremely high IL-1β concentrations (at least several ng/mL) which are more than 10–100-fold higher than actually measured in OA synovial fluid samples [13,14]. In the present study, we used OA-relevant IL-1β concentrations and observed an accelerated morphological shift with clearly visible and increased numbers of stress fibers after IL-1β treatment. This observation was described earlier in dedifferentiating chondrocytes [10], however, such an effect was not previously described for low IL-1β concentrations.

The gene expression profile of dedifferentiated chondrocytes has been studied previously by others, but only over much longer culture periods [5,32]. Gene expression levels of analyzed genes in the present study were only influenced when IL-1β was present in the treatment medium. One reason for unchanged COL1A1, COL10A1, and ADAMTS5 expression might be that OA chondrocytes are already IL-1β-primed due to the pro-inflammatory milieu in the synovial fluid of OA patients [13,14] and these three genes are not susceptible any more for IL-1β treatment. On the other hand, it is possible that these genes were very responsive to monolayer culture conditions and the observed gene expression levels had already been achieved at very early time points, and therefore no treatment was able to induce further changes. In the case of SOX9, COL2A1, COMP, and ACAN, the observed decrease in gene expression after IL-1β treatment was in accordance with earlier studies using hyperoxic conditions and with treatments with much higher IL-1β concentrations [6,10]. Concomitantly with SOX9, COL2A1, COMP, and ACAN inhibition, low-dose IL-1β treatment resulted in upregulation of MMP13 gene and in unchanged COL10A1 and ADAMTS gene expression levels, suggesting that IL-1β not only downregulates several hyaline cartilage-specific ECM genes, but also perpetuates ECM degradation by preserving high expression levels of distinct matrix-degrading enzymes [12].

In order to confirm our findings at the protein level, selected ECM molecules were stained histologically and immunohistologically. As expected, IL-1β strongly inhibited sGAG deposition in line with the above-mentioned ACAN gene expression data and as known for long-time dedifferentiation cultures [6,10]. In contrast to our PCR results, COMP staining intensity was not affected by IL-1β treatment, and type X collagen staining was more intense in IL-1β-treated groups. One might argue that the decrease in COMP gene expression was initiated shortly before chondrocyte harvest at day 7 and the changes were not yet translated to the protein level, and that COMP protein could equally accumulate extracellularly during seven days physioxic culture (in contrast to hyperoxic conditions) [9]. The opposite might be the reason why type X collagen staining was stronger in IL-1β-treated groups. IL-1β might affect COL10A1 immediately after chondrocyte isolation followed by quick but intense type X collagen protein synthesis, but after one or two days COL10A1 expression decreases due to monolayer culture conditions, which seem to have a strong effect on COL10A1 and could not be modulated by IL-1β [10,34]. It is well known that extracellular matrix proteins are characterized by an extended half-life and slow turnover at the protein level [39,40]. On the one hand, this fact can explain why expression data at the RNA and protein level do not always match. On the other hand, this underlines the importance of analyzing expression at both levels to understand the full picture. This might also explain why no differences were found at the gene expression level of COL10A1. NE did not exhibit any effect or modulate the effects of IL-1β in our monolayer chondrocyte culture, either at the gene nor at the protein expression level, although the ERK1/2 signaling pathway was activated shortly after NE treatment in freshly isolated chondrocytes as described earlier [38,41], and although Lorenz et al. observed the NE-mediated reversion of IL-1β effects on MMP-13 or GAGs in three-dimensional culture [19]. One reason that could explain this surprising phenomenon might be that the expression profile of ARs in chondrocytes massively changes during seven days, and due to NE and IL-1β treatment. In fact, in NE-treated chondrocytes expression of all detected ARs decreased markedly. Downregulation of ARs by NE was already shown in aorta muscle cells, hamster ovary cell lines, and in HT29 cell line (human colon cancer cell line) [42,43,44], but the inhibitory effect of NE on AR gene expression in human articular chondrocytes was shown for the first time in the present study. Similar dramatic inhibition of AR expression was observed in IL-1β-treated groups, where all ARs disappeared at mRNA level. Bucher et al. observed that pro-inflammatory cytokines downregulate α1-ARs in all organs of rats during endotoxemia and Koto et al. found deceased β-AR expression in rat tracheal and bronchial smooth muscle cells after IL-1β treatment [45,46]. Thus, it is highly likely that, besides NE, IL-1β is also responsible for AR downregulation in our OA chondrocyte culture. In contrast to earlier studies demonstrating that IL-1β (1 ng/mL) strongly reduced IL-1βR expression level in human articular chondrocytes [47], IL-1βR expression remained unchanged in our OA chondrocyte culture. Neither NE nor IL-1β alone or combined influence IL-1βR expression, which leads to long-term IL-1β influence without any limitation and might explain also the dominance of IL1-dependent/induced effects. Naldini et al. observed enhanced expression and release of IL-1βR in human peripheral blood mononuclear cells cultured under 2% O_2_ concentration for 16–40 h [48]. Thus, physioxia seems to act as an antagonist of IL-1β and might stabilize IL-1βR expression in our OA chondrocyte culture.

Aging is one of the major risk factors for OA development [49]. With increasing age, the OA-specific loss of articular chondrocyte phenotype is accelerated [50,51]. The sex steroid estrogen has also been shown to influence chondrocyte homeostasis by inducing the expression of specific chondrogenic markers such as type II collagen, and inhibiting catabolic cytokine and MMP expression [52]. However, none of our tested parameters were regulated to be age- or gender-dependent, either with untreated nor with NE- or IL-1β-treated chondrocytes. The reason for this might be the low variance of age of the analyzed patients and the fact that most of the female patients were already in the postmenopausal stage of life.

In conclusion, this study demonstrated that low-dose IL-1β is a strong inducer of chondrocyte dedifferentiation even in short-term culture and under physioxic conditions. In contrast and unexpectedly, NE did not exhibit any effect on monolayer chondrocytes, in either low or in high concentrations, even though relevant receptors were present. NE was also not able to modulate the effects of low-dose IL-1β. Thus, the very low inflammatory status obviously exerts a dominant effect which massively contributes to the chondrocyte dedifferentiation process during OA pathogenesis and should therefore be targeted early and primarily in OA therapy.

## 4. Materials and Methods

### 4.1. Human Articular Chondrocyte Isolation and Culture

Human articular cartilage samples were obtained from OA patients undergoing total knee replacement surgery. The samples were anonymized, thus no approval by the Ethics Committee of the Goethe University Frankfurt was necessary. All experiments were performed in accordance with relevant guidelines and regulations. The experimental cohort included 10 patients (patient information is given in Table 1).

Macroscopically normal-looking cartilage tissue was removed from the subchondral bone, cut into pieces approximately 1 × 2 mm in size, and digested with sterile filtered 0.2% (*w*/*v*) pronase (Roche) in Dulbecco’s modified Eagle medium (DMEM)/F12 (Gibco, Thermo Fisher Scientific, Darmstadt, Germany) containing 1% penicillin/streptomycin (P/S, Gibco) for 2 h on a shaker at 37 °C and at 60 rpm. Then, cartilage pieces were washed three times with 1× DPBS and digested with sterile filtered 280 U/mL collagenase II (Biochrom, Berlin, Germany) in DMEM/F12 overnight on a shaker at 37 °C and at 60 rpm. After the digestion steps, the chondrocytes were passed through a 70 mm nylon mesh (Falcon, VWR, Darmstadt, Germany) to remove residual cartilage fragments, centrifuged at 300× *g* for 5 min, resuspended in DMEM/F12 supplemented with 10% fetal calf serum (FCS, Sigma-Aldrich, Munich, Germany) and 1% P/S, and seeded in a density of 20,000 cells/cm^2^ into T75 cell culture flasks and cultured in a humidified atmosphere at 37 °C and in 2% O_2_/5% CO_2_ overnight to allow attachment of cells.

### 4.2. Chondrocyte Stimulation

After overnight attachment, chondrocytes were treated with different concentrations of NE (10^−8^ M or 10^−6^ M representing α-AR- or β-AR-dominant concentrations, Sigma-Aldrich) and/or IL-1β (0.5 ng/mL, OA-specific IL-1β concentration, Peprotech, Hamburg, Germany) in DMEM/F12 supplemented with 10% FCS, 1% P/S, and ascorbic acid (Sigma-Aldrich). Chondrocytes were cultured for seven days with NE and/or IL-1β at 37 °C and in 2% O_2_/5% CO_2_. Within seven days, chondrocytes started to dedifferentiate without reaching a fully dedifferentiated status, allowing possible manipulation by the distinct treatments. Untreated chondrocytes served as the control group and day 0 chondrocytes as starting point controls. Cell culture medium with freshly diluted supplements was changed on day four. The same experimental setup was performed in chamber slides for histological investigations. After seven days, chondrocyte morphology was documented microscopically and after detachment, cells were counted and frozen at −80 °C for analysis. Chondrocytes in chamber slides were fixed using 4% PFA (Thermo Fisher Scientific, Darmstadt, Germany) for histological investigations. In addition, supernatants were harvested and immediately used for cell viability assay.

### 4.3. NE-Dependent Signal Transduction

In order to examine whether monolayer chondrocytes respond to NE, the two major AR-dependent signaling pathways—the phosphorylation of PKA and ERK1/2—were investigated. Overnight, attached chondrocytes were treated for 15 min with NE (10^−8^ or 10^−6^ M). Protein isolation was performed using NucleoSpin RNA/Protein kit (Machrey Nagel, Düren, Germany). Samples were loaded onto 10% SDS-PAGE (sodium dodecyl sulfate polyacrylamide gel electrophoresis) and electrotransferred to a polyvinylidene difluoride (PVDF) membrane. Membranes were blocked with 5% bovine serum albumin for 1 h at room temperature before incubation with primary antibodies for total ERK (#9107; Cell Signaling Technology, Frankfurt/Main, Germany), phosphorylated ERK (#4370; Cell Signaling Technology), total PKA (#32514; Abcam, Cambridge, United Kingdom), phosphorylated PKA (#32390; Abcam), and GAPDH (#MA5-15738; Thermo Fisher Scientific) at 4 °C overnight. The membranes were washed with TBST and incubated with HRP-conjugated secondary antibody (DAKO, Hamburg.Germany) for 1 h at room temperature. The target protein expression was detected using the chemiluminescence (ECL, Scientific, Darmstadt, Germany) reagents, with GAPDH as the endogenous control.

### 4.4. Morphological Analyzes

Chondrocyte cellular morphology, which is directly linked to cell metabolism and ECM synthesis [24] was examined by phase-contrast microscopy as well as staining of the actin cytoskeleton at day 7 of dedifferentiation. For actin staining, fluorescent-labeled phalloidin was used according to manufacturer’s instructions (FAK100 Actin Cytoskeleton/Focal Adhesion Staining Kit, Merck, Darmstadt, Germany) followed by documentation at a fluorescence microscope (Nikon, Minato, Japan).

### 4.5. Determination of Cell Viability

To demonstrate that treatment with NE and IL-1β has no cytotoxic effects, LDH assay (Takara) was performed according to manufacturer’s instructions using cell culture supernatants from day 7, cell culture medium without cells, and the dead-control (300 µL Triton-X per T75 for 15 min). Supernatants were analyzed in a 96-well plate as duplicates in a plate reader.

### 4.6. RNA Isolation and PCR

RNA isolation from monolayer chondrocyte pellets was performed using NucleoSpin RNA/Protein kit (Machrey Nagel, Düren, Germany) according to the manufacturer’s instructions. Synthesis of cDNA was performed using qScript cDNA Supermix (Quanta Biosciences, VWR, Darmstadt, Germany). For gene expression analysis of different AR subtypes and the IL-1β receptor (IL-1βR) on chondrocytes at day 0 and day 7, reverse transcription PCR was used (Taq PCR Master Mix kit, Qiagen, Hilden, Germany). PCR products were run on a 1.8% (wt/vol) agarose gel, stained with GelRed Nucleic Acid Gel Stain (Biotium, Fremont, CA, USA). In addition, TH gene expression was quantified in order to consider possible autocrine effects. GAPDH served as housekeeping gene. In addition, gene expression changes of ARs and ECM-related genes (SOX9; COL1A1; COL2A1; COL10A1; COMP, ACAN; MMP13; ADAMTS-4; ADAMTS-5) after seven days of monolayer culture were analyzed using Quanta PerfeCta SYBR Green FastMix (Quanta Biosciences, VWR, Darmstadt, Germany) in qTOWER3 Thermocycler (Analytik Jena, Jena, Germany). Relative gene expression was determined by the ∆∆Ct method using qPCR3.2 software (Analytik Jena) [52]. Human RPII served as housekeeping gene [53,54]. All primers were synthesized by Thermo Fisher Scientific (Table 2).

### 4.7. Cytological Staining

Chondrocytes incubated in chamber slides were stained for ECM components to analyze the effects of NE and/or IL-1β treatment. The metachromatic dye DMMB (0.1% 1,9-dimethyl-methylene blue, Sigma-Aldrich) in dd. H_2_O was used to detect newly synthesized sGAG. COMP and type X collagen were stained using polyclonal rabbit primary antibodies (COMP: rabbit anti-COMP 4-1 (Immundiagonstik AG, Bensheim, Germany) [55], 1:100; type X collagen: ab58632, Abcam, Cambridge, United Kingdom, 1:200) and ImmPRESS™ peroxidase polymer goat anti-rabbit IgG biotin secondary antibody (Linaris, Dossenheim, Germany). Staining was visualized using Vectastain ABC-AP detection Kit (Linaris).

### 4.8. Statistical Analysis

All experiments were carried out with cells of 4–10 patients. Data were presented as box plots with medians. For statistical evaluation of different groups to the day 0 control group, Wilcoxon signed-rank test (to hypothetical value 1) was used. Comparisons between treatment groups were carried out using one-way ANOVA/Student–Newman–Keuls post hoc test or the non-parametric Mann–Whitney *U* test and Bonferroni post hoc test. *p* values less than 0.05 were considered significant. All statistical analyzes were performed using SigmaPlot 14.0 software.

## Figures and Tables

**Table 1:** Characteristics of patients in study.

Patient Characteristics	Number/Mean Age ± SEM
Total (number/age)	10/65.33 ± 11.77
Female (number/age)	6/64.83 ± 12.06
Male (number/age)	4/66.33 ± 11.09

**Table 2:** The primers used for PCR.

Gene Name	NCBI Reference	Forward (5′–3′)	Reverse (5′–3′)
GAPDH	NM_001289745.2	CTCCTGTTCGACAGTCAGCC	TTCCCGTTCTCAGCCTTGAC
TH	NM_000360.3	CAGGCAGAGGCCATCATGT	GTGGTCCAAGTCCAGGTCAG
ADRA1A	NM_000680.3	CCATGCTCCAGCCAAGAGTT	TCCTGTCCTAGACTTCCTCCC
ADRA1B	NM_000679.3	GTCCACCGTCATCTCCATCG	GAACAAGGAGCCAAGCGGTAG
ADRA1D	NM_000678.3	TGACTTTCCGCGATCTCCTG	TTACCTGCCACGGCCATAAG
ADRA2A	NM_000681.3	TGGTCATCGGAGTGTTCGTG	GCCCACTAGGAAGATGGCTC
ADRA2B	NM_000682.6	GACATTTCACCGGCAACACC	GGGACTGAGAACCAGGAAGC
ADRA2C	NM_000683.3	CGATGTGCTGTTTTGCACCT	GGATGTACCAGGTCTCGTCG
ADRB1	NM_000684.2	TAGCAGGTGAACTCGAAGCC	ATCTTCCACTCCGGTCCTCT
ADRB2	NM_000024.5	CAGAGCCTGCTGACCAAGAA	GCCTAACGTCTTGAGGGCTT
ADRB3	NM_000025.3	GCCAATTCTGCCTTCAACCC	GCCAGAGGTTTTCCACAGGT
IL1R1	NM_000877.4	AGGGATGACTACGTTGGGGA	CTCCAGCTCAAGCAGGACAA
POLR2A	NM_000937.5	GACACAGGACCACTCATGAAGT	GTGCGGCTGCTTCCATAAG
COL1A1	NM_000088.3	ACGTCCTGGTGAAGTTGGTC	ACCAGGGAAGCCTCTCTCTC
COL2A1	NM_001844.4	TTCAGCTATGGAGATGACAATC	AGAGTCCTAGAGTGACTGAG
COL10A1	XM_011535433.3	CCCTCTTGTTAGTGCCAACC	AGATTCCAGTCCTTGGGTCA
COMP	NM_000095.2	AGGGAGATCGTGCAGACAA	AGCTGGAGCTGTCCTGGTAG
SOX9	NM_000346.4	ACACACAGCTCACTCGACCTTG	AGGGAATTCTGGTTGCTCCTCT
ACAN	NM_001135.3	TCCCCTGCTATTTCATCGAC	CCAGCAGCACTACCTCCTTC
ADAMTS 4	NM_001320336.1	AGGGAAGGGGACAAGGACTA	TATCACCACCACCCTGGATT
ADAMTS 5	NM_007038.4	TACTTGGCCTCTCCCATGAC	TTTGGACCAGGGCTTAGATG
MMP13	NM_002427.4	GACTGGTAATGGCATCAAGGGA	CACCGGCAAAAGCCACTTTA

## Supplementary Materials

Supplementary materials can be found at https://www.mdpi.com/1422-0067/20/5/1212/s1.

## Abbreviations

AR	Adrenergic receptor
ACAN	Aggrecan
ADAMTS4	ADAM metallopeptidase with thrombospondin type 1 motif 4
ADAMTS5	ADAM metallopeptidase with thrombospondin type 1 motif 5
cDNA	Complementary desoxyribonucleic acid
COL1A1	Collagen type I alpha 1 chain
COL2A1	Collagen type II alpha 1 chain
COL10A1	Collagen type X alpha 1 chain
COMP	Cartilage oligomeric matrix protein
DA	Dopamine
DMEM	Dulbecco’s modified Eagle’s medium
DMMB	1,9-Dimethyl-methylene blue
DPBS	Dulbeccos phosphate-buffered saline
E	Epinephrine
ECM	Extracellular matrix
ERK	Extracellular signal-regulated kinases
FCS	Fetal calf serum
GAG	Glycosaminoglycan
GAPDH	Glyceraldehyde-3-phosphate dehydrogenase
HRP	Horseradish peroxidase
IL-1β	Interleukin-1 beta
IL1β-R	Interleukin-1 receptor type 1
L-DOPA	L-3,4-dihydroxyphenylalanin
LDH	Lactate dehydrogenase
MMP13	Matrix metallopeptidase 13
mRNA	Messenger ribonucleic acid
NE	Norepinephrine
OA	Osteoarthritis
PCR	Polymerase chain reaction
PFA	Paraformaldehyde
PKA	Proteinkinase A
PVDF	Polyvinylidene difluoride
RPII	RNA polymerase II subunit A
RT-PCR	Reverse-transcriptase PCR
sGAG	Sulphated glycosaminoglycans
SDS-PAGE	Sodium dodecyl sulfate polyacrylamide gel electrophoresis
SOX9	SRY-box 9
TBST	Tris buffered saline with Tween20
TH	Tyrosine hydroxylase
