# Supplementary material for: Role of Norepinephrine in IL-1β-Induced Chondrocyte Dedifferentiation under Physioxia
Source: Int J Mol Sci. 2019 Mar 11;20(5):1212. doi: 10.3390/ijms20051212 (PMC6429278; doi:10.3390/ijms20051212)
Supplement: Supplementary file 1 [file ijms-20-01212-s001.pdf]

Supplementary Material - ijms-416948

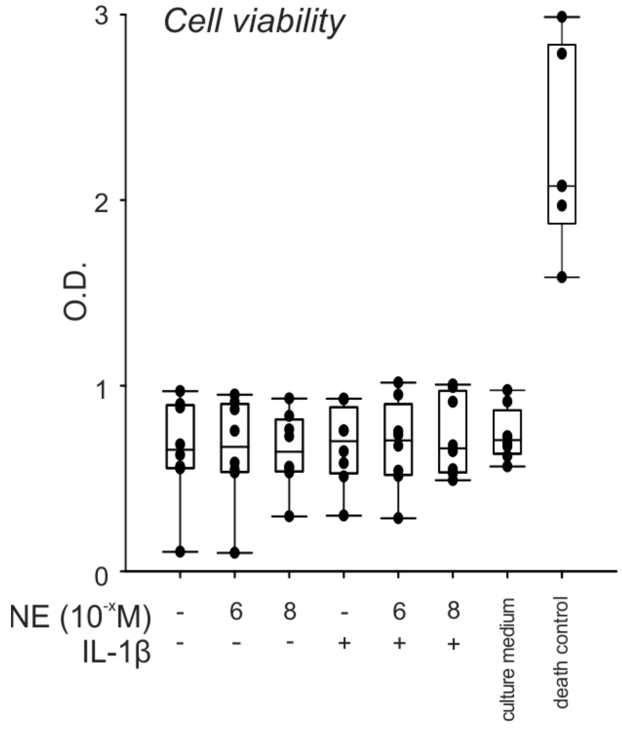

Figure S1. Effect of NE and IL-1β on chondrocyte viability. Lactate dehydrogenase (LDH) release in OA chondrocyte cultures at day 7 after treatment with NE, IL-1β, or NE + IL-1β. Each black circle represents cells from an individual patient (n=8). (O.D. – optical density)

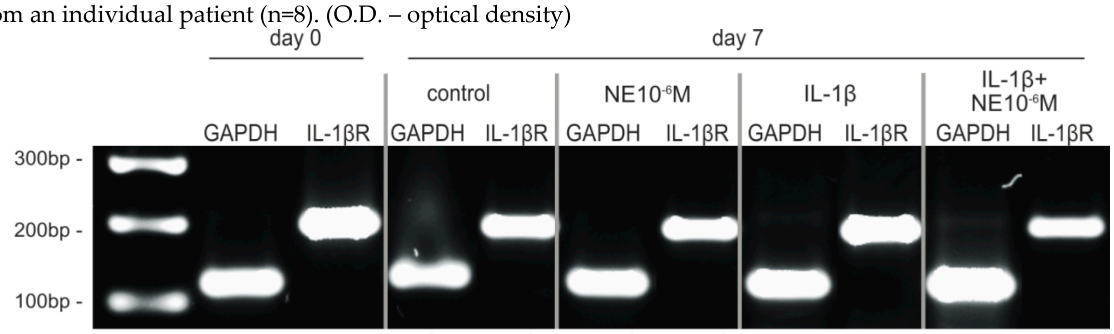

Figure S2. IL-1βR expression in OA chondrocytes. Expression of IL-1βR in untreated primary chondrocytes at day 0 and in NE- or/and IL-1β-treated at day 7 (representative images of cells isolated form one OA patient).
